# Age and gender differences in the value development of Dutch adults in 11 years of longitudinal data

**DOI:** 10.1016/j.jrp.2024.104540

**Published:** 2024-12

**Authors:** Oscar Smallenbroek, Adrian Stanciu

**Affiliations:** a Joint Research Centre, European Commission, Ispra, Italy; b Faculty of Humanities, Education and Social Sciences (FHSE), University of Luxembourg, Luxembourg

**Keywords:** Human values, Value development, LISS panel, Gender, Age

## Abstract

Value development over the life-span is rarely studied due to theory and data limitations. We use the LISS data, a Dutch longitudinal dataset, to study value change in adults aged 25–70 over 11 years from 2008 to 2019 (N=10,860), using the neo- socioanalyitcal model (NSM) as a theoretical framework. We find few cohort differences, differences between age groups and non-linear value change within individuals that continues until late adulthood. Gender differences in mean-levels are stable except in universalism and self-direction, while gender differences in rates of change are observed. We conclude that the NSM provides a fruitful framework to interpret value change as a maturation process toward becoming functioning members of society along gendered and age-graded normative stages.

## Highlights

• Value change is social focused in mid-adulthood.
• Universalism increases in relative importance throughout the life-span.
• Few cohort differences; GDP and unemployment effect value ratings and preferences.
• Large gender differences in relative importance of values formed before age 25.
• Men and women change values at similar rates leading to convergence in ranking.

## 1 Introduction

Values are known to form during childhood socialization and transitions into adolescence and young adulthood (Bardi et al., 2009, Cieciuch et al., 2016, Vecchione et al., 2019, Pöge, 2020). During these processes gender differences in value preferences emerge (Milfont et al., 2016, Schwartz and Rubel, 2005) and gender specific trajectories of value development can be observed (Cieciuch et al., 2016, Lönnqvist et al., 2018, Vecchione et al., 2019). However, there is ambiguity in the literature with respect to what happens during adulthood and subsequently (25 years old or older). Researchers either assume there is negligible change in values during adulthood and therefore interpret differences in values between individuals as cohort changes (Inglehart, 1977) or assume values change over the lifespan and interpret differences between individuals as lifespan changes happening within individuals (Dobewall et al., 2017, Gouveia et al., 2015, Robinson, 2013, Vilar et al., 2020). The present paper contributes to resolving this inconsistency in the literature (Bardi and Goodwin, 2011, Milfont et al., 2016, Leijen et al., 2022, Smallenbroek et al., 2023) with evidence from a long-running longitudinal panel study in the Netherlands.

The assumption of value stability in adulthood draws on the formative years hypothesis (Inglehart, 1977) which claims cohort replacement is the main driver for value change in society. This assumption is however challenged by recent theoretical developments that describe mechanisms leading to lifespan changes in values (Bardi & Goodwin, 2011). One recent theoretical model – the Neo-Socioanalytical Model of personality development (NSM) (Caspi et al., 2005, Roberts and Nickel, 2017) – describes how personality can change across the lifespan due to intervening biological and socio-cultural factors. The NSM posits that values are likely to change according to identity and social role investments which unfold along a normative lifespan, where the overall direction of change is towards becoming a functional member of society.

Additionally, longitudinal data supports the notion that values can change in adulthood (Schuster et al., 2019). For example, it has been documented that major events such as the COVID-19 pandemic, (Bojanowska et al., 2021, Daniel et al., 2021, Vecchione, 2022), terrorist attacks (Verkasalo et al., 2006), the 2008 financial crisis (Sortheix et al., 2019) and life transitions such as migration and childrearing (Bardi et al., 2014, Lönnqvist et al., 2018) can result in changes in values. However, these studies focus on transitions or events, thus studying a narrow interval in the lifespan. The existent studies are ill-equipped for documenting long-term changes in values. The present paper addresses value development as changes in value preferences in a population involving changes within people over the lifespan (see also Bardi and Goodwin, 2011, Milfont et al., 2016, Leijen et al., 2022, Smallenbroek et al., 2023) following the definitions of values in the Theory of Basic Human Values (Schwartz, 1992).

We contribute to the literature with a study of value development in a representative sample of the adult population in the Netherlands spanning 11 years from 2008 to 2019. We use the LISS panel data as did Leijen et al. (2022) but the present study differs methodologically in terms of scale construction, modeling strategy and sample size. We therefore assess whether their results are replicable with a different dataset and methodology while extending their work by including controls for the economic conditions, interpreting the results within the NSM and focusing on rates of change and gender differences in these rates of change.

Considering value measurement, the present study is based on a careful analysis of the theoretical fit and predictive validity of the questionnaire items in the LISS data conducted by Leijen and colleagues (Smallenbroek et al., 2024). In terms of the sample, Leijen et al. (2022) used only information from participants who participated in every assessment from 2008 to 2020 (*N*=1,599) thus there is a danger that their findings are biased by sample attrition. For example, individuals with strong social values may be intrinsically motivated to participate in a panel study. Meanwhile, the present study uses all information available thus retaining a higher degree of generalizability to the Dutch population. The present research uses a sample containing all participants who had no missing data on all value scales (*N*=10,860). Additionally, we restrict the age range to individuals 25–70 to focus on the adult lifespan while Leijen et al. (2022) include a wider age range 16–83, which also captures the more volatile period of adolescence.

Regarding methodology, our multilevel modeling approach allows a more precise measure of time, in months instead of years, while the larger sample size allows us to distinguish ten cohort groups (instead of four). A second difference is the flexibility with which we modelled rates of change longitudinally. Whereas Leijen et al.’s (2022) methodology restricted rates of change to be similar across the lifespan by centering age on the cohort, we took a more flexible approach in that we modelled random rates of change as a function of the age at entry to the panel. In this manner the rates of change are independent of the cohort, as several refreshment samples include observations of respondents of different cohorts entering the panel at the same age.

Finally, compared to Leijen et al. (2022), we exclude data collected at the onset of the COVID-19 pandemic (2020) as this societal event might have affected the lifespan value development (Bojanowska et al., 2021, Daniel et al., 2021, Vecchione, 2022). Neither the available data nor the existent methodological tools suffice in separating the impact of period effects like COVID-19 from cohort and ageing effects. Moreover, we include data on the GDP growth rate and unemployment to account for the economic crisis that unfolded during the start of the panel in 2008 (Centraal Bureau voor de Statistiek, 2024). In terms of interpretations of results, we frame the present analyses within the NSM and focus on gender differences. Given that identity and social roles are profoundly shaped by gender throughout the lifespan (Ridgeway, 2009), we expect gender to also shape value development in adulthood.

This paper will present mean-level changes and intra-individual rates of change in personal values of men and women aged 25–70 in cohorts born from 1936 to 1995. We can observe that values do change within individuals across ages 25–75, that these changes are more pronounced in the younger age groups and differ slightly between men and women. Additionally, we observe differences in values and changes in value priorities between respondents of different ages, even when controlling for cohort effects and the economic crises.

### 1.1 Value theory

The Theory of Basic Human Values (TBHV) states that values represent socially desirable goals that people pursue in coping with a limited set of existential, psychological and social needs (Schwartz, 1992, Schwartz et al., 2012). Accordingly, there is a finite number of ways in which individuals can resolve these needs, which are addressed by the motivations and goal content of values. The set of values in the TBHV are universal, as all individuals are assumed to have the same set of needs. The values are universalism, benevolence, conformity, tradition, security, power, achievement, hedonism, stimulation, and self-direction (see Appendix, Table 12 for definitions).

**Fig. 1:**
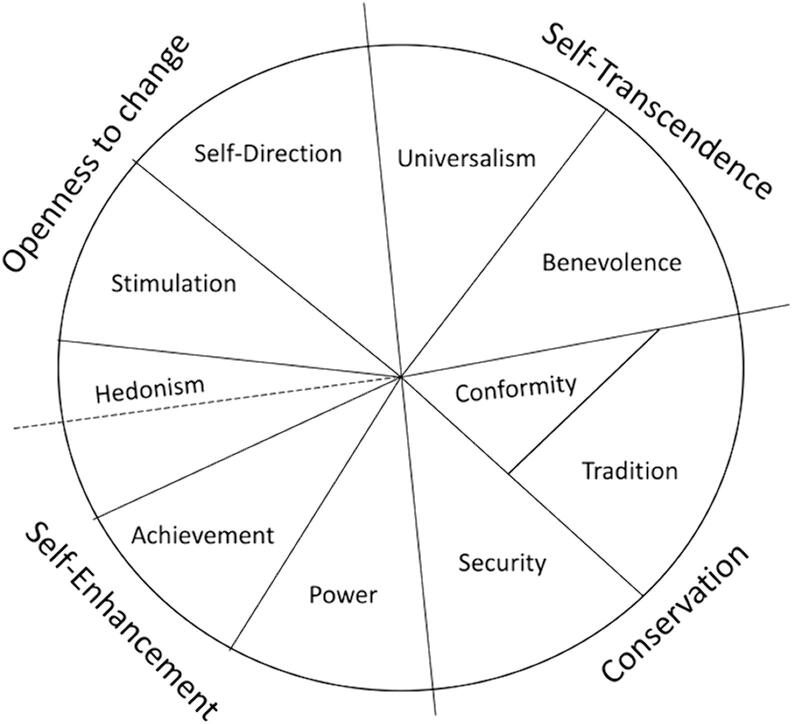
Circular structure of human values in the Theory of Basic Human Values.

The TBHV proposes that values are organized in a circular structure depending on the degree of (in)-compatibility in their goals and motivations (see Fig. 1). For instance, values that share goals can be summarized by a higher-order value. For example, security, conformity, and tradition all share the goal of maintaining the status quo and together form the higher-order value of conservation. The higher-order value of openness to change is composed of hedonism, stimulation, and self-direction values. Universalism and benevolence values create the higher order value of self-transcendence – that is to care for others, society and environment. Lastly, the self-enhancement values are achievement and power. However, there are also value conflicts. Universalism opposes power since the motivational goal of the former is protection for the welfare of all people and environment while the latter is dominance over people and resources. Conformity conflicts with self-direction since the motivational of the former is restraint of impulses likely to violate social expectations or norms and of the latter is independent thought and action. The incompatibilities between goals and motivations present several tradeoffs for individuals through which values guide decision making; individuals must choose one set of values as more important than another.

### 1.2 Value development in adulthood

#### 1.2.1 Theory

Roberts et al. (2008) organized the literature on personality development across the lifespan into the Neo-Socioanalytic Model (NSM; Roberts & Nickel, 2017). The NSM sees values as the motivational layer of personality which changes throughout the lifespan. The mechanisms, direction, and shape of development is captured in eight principles. Of these, four are empirically well-documented. The cumulative-continuity principle describes an increasing rank-order consistency of values with age, with a plateau or decrease after the age of 60. This principle implies that personality change is ongoing in adulthood. The maturity principle describes that people become more socially dominant, agreeable, conscientious and emotionally stable with age. In this case, maturity is defined from a functionalist perspective, as being able to function, contribute and engage with society. Third, the plasticity principle states that the environment can modify values at any age. Fourth, the social investment principle states that investments in social roles and identities are the drivers of personality change and contribute to the maturation process.

#### 1.2.2 Evidence

Most of the evidence on value development in adults is from cross-sectional studies (Dobewall et al., 2017, Vilar et al., 2020, Fung et al., 2016, Schwartz, 2015, Robinson, 2013). Cross-sectional research, however, is poorly equipped for distinguishing between effects due to cohort, the ageing process, and specific life events. Only a handful of studies has looked at value development of adults from a longitudinal perspective (see Schuster et al., 2019). The prevalent evidence is limited to brief periods in the lifespan, with observation intervals ranging between three months (Study 3, Bardi et al. 2009) and three years apart (Milfont et al., 2016). In addition, the evidence is predominantly based on samples of convenience, which makes it difficult to generalize to the general population. To our knowledge, there are only two studies with a true lifespan approach to value development in adulthood. In two studies, Smallenbroek et al. (2023) examined value development over 18 and 26 years respectively of longitudinal data from German adults. Leijen et al. (2022) investigated a 12-years period in the lifespan of Dutch adults. Milfont et al. (2016) studied value development in adults (from New Zealand) however their longitudinal data was limited to a three-year interval. Below we summarize the findings of the cross-sectional and longitudinal studies.

**Age Effects.**Smallenbroek et al. (2023) studied value development in German adults in longitudinal data from a convenience sample followed throughout 1999–2017 and a sample representative of the population interviewed during 1990–2016. Smallenbroek et al. (2023) found changes in value priorities become less pronounced with age. Their results show that self-enhancement values gradually become less relevant whereas self-transcendence and conservation values become more important corroborating past evidence suggesting that people become increasingly socially oriented with age. Moreover, they were the first to show that changes in the importance people attribute to openness to change values over time are curvilinear instead of linear as previously thought. Thus, examining narrow periods in the lifespan may limit our understanding of value developmental over the lifespan.

Leijen et al. (2022) used data from the LISS panel and examined intra-individual value change relative to other values within cohorts observed throughout 2008–2020 in the Netherlands. They found individuals increased the importance of universalism and self-direction over time, with a larger increase in Millenials. Millenials were the only generation which increased benevolence values. Stimulation values decreased within individuals for all cohorts except the oldest while the decrease was fastest in Millenials. Hedonism and achievement values did not change within individuals. Security values increased within individuals except for the oldest cohort. Conformity values did not change within individuals.

In terms of differences between cohorts and ages, Leijen et al. (2022) found the youngest cohort to value benevolence less than the oldest generation. Each generation scored universalism lower than the previous cohorts. Thus, there was a generational shift towards lower self-transcendence values. Self-direction values were less important for younger cohorts when comparing those born after 1965 (Generations X and Millennials) than those born before. However, each younger cohort valued stimulation more than the previous cohort. Thus, in terms of openness to change values, there were conflicting cohort trends. Each generation prioritized achievement less compared to the oldest generation (1925–45) and younger generations preferred security less than older generations. In terms of differences between ages within cohorts, Leijen et al. (2022) found older individuals valued universalism, conformity and security more while they valued stimulation and hedonism less. Milfont et al. (2016) studied value development using value ratings in a representative sample with an age range from 25 to 75 observed throughout 2009–2012 in New Zealand. They included up to a third polynomial of age with an interaction with gender but did not account for cohort effects. In terms of intra-individual change, they found that respondents became less conservative and that the change was steeper for older respondents. Individuals also became less open to change over time with the slope being steepest from age 25–40. In terms of self-transcendence values, they also observed an average decrease in preference within individuals. The decrease was larger for those between 25 and 40 and after age 65. On average, the preference for self-enhancement values decreased and more so for older individuals.

In terms of differences between individuals of different ages, Milfont et al. (2016) found self-transcendence and conservation values to be rated higher in older individuals while openness to change values were rated lower and there was no difference in self-enhancement values. They also found moderate stability in value preferences over a three-years period exempting conservation values which displayed lowest stability at younger ages (25–34) and highest stability at older ages (67–73).

Short longitudinal studies echo this evidence as they also report relative stability in value development, with lower stability observed at larger time intervals. Test-retest correlations were on average *r* = 0.75 for a six-week interval (Schwartz, 2005) and *r* = 0.56 for intervals between one year and two years (Lönnqvist et al., 2018, Bardi et al., 2014). In addition, these types of studies also find that age influences value preferences of people. Age is positively associated with a preference for values of conservation and self-transcendence but negatively with a preference for values of openness-to-change and self-enhancement (Schwartz, 2005, Robinson, 2013, Gouveia et al., 2015).

**Gender Differences.** There are observable gender differences in value profiles (Lyons et al., 2005; Döring et al., 2010) and value curves (Cieciuch, Davidov, & Algesheimer, 2016) in the initial developmental stages. It is still unclear how gender differences develop in adulthood, as the longitudinal evidence is limited. Both Leijen et al. (2022) and Milfont et al. (2016) found that women value self-transcendence and conservation more than men while they value openness to change and self-enhancement less than men. Gender differences were addressed also in cross-sectional correlational studies and the findings are in tune with the two longitudinal studies (Schwartz & Rubel, 2005). Men seem to prioritize values of self-enhancement and openness-to-change more than women. Meanwhile, women prioritize values of self-transcendence and conservation more than men.

Even less documented is the question whether value developmental trajectories unfold similarly between men and women throughout the lifespan. Leijen et al. (2022) found gender differences in the intra-individual change over the studied 12-years period for benevolence, security and conformity values. Men increased their ratings of these social focused values faster than women and decrease the importance of hedonism values while women do not show a statistically significant rate of change. Meanwhile, Milfont et al. (2016) found differences in the value developmental trajectories between men and women only in conservation values (security and conformity).

### 1.3 The present research

Despite a long-standing assumption of value stability after the age of 25 (Schwartz, 1992, Rokeach, 1973) the recent evidence would suggest otherwise (Vecchione et al., 2016, Smallenbroek et al., 2023, Leijen et al., 2022). We expect values to develop over an 11-year observational period. In terms of average gender differences, we hypothesize that adult men will always score higher on values of self-enhancement and openness-to-change than women while adult women will always score higher on values of self-transcendence and conservation than men (hypothesis 1). These differences are likely partly due to the internalization of gender stereotypes and social roles into identity which then lead to choices, made within differential opportunity structures for men and women and cultures of sexism (Bareket and Fiske, 2023, Belingheri et al., 2021), leading to environments that re-inforce and reflect gender differences in motivation and personality (Cech, 2013, Ellemers, 2018, Ridgeway, 2009). Values may therefore also change at different speeds depending on gender (hypothesis 2) as both men and women are exposed to daily reminders of their gender throughout the lifespan thus shaping, through repeated exposure and reinforced learning (Kolb et al., 2001, Martin and Ruble, 2010), the value motivational contents acquired during childhood (Cieciuch et al., 2016, Döring et al., 2010). These processes would fit into the social investment principle of the NSM which claims people commit to normative social roles existing in teenagerhood and young adulthood (Roberts & Nickel, 2017, pp. 163-164) and the identity development principle of NSM, which suggests that people invest in and commit to their identities throughout their lifespan resulting in increased personality differences with age (Roberts & Nickel, 2017, pp. 166-167).

On the other hand, men and women's value development may be similar. This hypothesis draws on the maturity principle of NSM. The maturity principle suggests that people adapt to function smoothly in society with age, in terms of personality traits these include being agreeable, conscientious, and emotionally stable (Roberts & Nickel, 2017, p.163). Agreeableness has been found to be positively related to universalism, benevolence, conformity and tradition and negatively associated with power while conscientiousness is associated with higher achievement, conformity and security, however emotional stability is unrelated to values (Parks-Leduc et al., 2015). Additionally, research shows people seek social and emotional stability with age suggesting that perceptions of death, endings in a more general sense, prompt community embeddedness in people (Löckenhoff & Carstensen, 2004). Thus, a further hypothesis is that both men and women become more socially oriented with age (Hypothesis 3). An open question is whether gender differences in values converge towards social focused values or whether gender differences in values remain stable.

We expect changes in values to slow down with age (hypothesis 4). Roberts & Nickel (2017) describe consistent findings of increasing rank order stability up to age 60, which they call the cumulative-continuity principle. Additionally, Erik Erikson argued that the developmental stage in adulthood is characterized by a dynamic process between generativity (i.e., creation) and stagnation (i.e., stability) (Erikson, 1997). As adults, people invest in and commit to their social roles while the primary drive at this developmental stage is to maintain what has been generated, for example, family or career. Resulting in an increased consistency with age in view of a person’s identity involving one’s values as well.

## 2 Method

### 2.1 Data

We used the Longitudinal Internet Studies for the Social sciences (LISS, https://www.lissdata.nl/about-panel), a panel study started in 2008 based on a true probability sample of households from the Dutch population register (Scherpenzeel & Das, 2010). LISS panel members are surveyed monthly via the internet whereas a module on personality including questions on values is administered annually. We used data up to 2019 to avoid any influences attributable to the COVID-19 pandemic and included all respondents aged 25–70 born from 1937 to 1994 who provided enough data on all value scales at least once. The analyses involved data from *N*=10,860 respondents (see for further details the Appendix). On average, respondents were observed on 3.1 occasions with a maximum of 8 observation points spanning 11 years.

### 2.2 Measures

***Values*.** Values were measured with the Rokeach Value Survey (RVS; Rokeach, 1973), the predecessor of the Schwartz Value Survey (SVS). Participants were asked to rate 34 items in view of how they felt those acted as guiding principles in their life using a 7-point Likert scale with anchors ranging from 1—*extremely unimportant* to 7—*extremely important*. Smallenbroek et al. (2024) were able to reproduce seven of the values described in the TBHV, namely universalism, benevolence, conformity, achievement, hedonism, stimulation and self-direction (see Table 1). For the purpose of this paper, the scales with at least three items were subjected to a multi-group confirmatory factor analysis (MGCFA) which constrained item loadings and intercepts to be equal across survey waves and confirmed measurement invariance across time (Hu & Bentler, 1999). The model parameters and fit indices of the MGCFA are shown in Appendix Table 4 and 5. We averaged the scores on the items and then subtracted the average of the all items for that person-year as described in Smallenbroek et al. (2024). This is the recommended and customary approach to measure value’s relative importance to an individual and is also known as ipsatization (Schwartz, 1992). We also report all results using ratings in the Appendix Table 10 and 11.

**Table 1:** Values and Corresponding Items.

Value	Item 1	Item 2	Item 3	Item 4
Universalism	a world at peace	freedom	equality	−
Benevolence	sincere and truthful	helpful	loving	open
Conformity	proper	polite	obedient	−
Achievement	a sense of accomplishment	social recognition	intellectual	−
Stimulation	a world of beauty	an exciting life	−	−
Hedonism	a comfortable life		−	−
Self-direction	creative	wisdom	−	−

***Socio-demographics:*** Respondents’ gender was coded as male = 0 or female = 1. They provided their age (*M*=49.16, *SD*=12.74, range = 25–70) at the time of survey and their year of birth (*M*=1963.03, *S.D*=13.03, range = 1937–1994). We standardized the age variable and used the year of birth to construct 10 indicator variables coded 1 if a respondent was born within the year range and 0 otherwise. Each cohort was 7.62 % to 11.25 % of the sample using as starting years: 1936, 1946, 1950, 1953, 1957, 1961, 1964, 1969, 1973, 1978, and 1983.

***GDP growth and unemployment rate*:** The quarterly GDP growth rate and the seasonally adjusted unemployment rate (Central Bureau of Statistics Netherlands, 2024) were matched with the year-month of the interview.

### 2.3 Analytical strategy

We modeled the differences in values between ages, genders and cohorts as well as the intra-individual change over time using multilevel growth models. Note that multilevel growth models and latent growth models provide identical estimates in their basic form (Chou et al., 1998), as used in this paper. We opted for the multilevel models as these can model time as a continuous variable, and therefore take into account the exact amount of time elapsed between observations. In contrast, latent-growth models require the time elapsed between two observations-points to be similar for all respondents. This was an important consideration as the personality module of the LISS data was fielded with considerable variability, the time elapsed between two observations ranged from 6-18 months.^1^ As a result, we operationalized time as the number of years since respondents entered the panel.^2^ Table 7 in the Appendix shows the n^th^ time a respondent was observed in the panel (rows) by the time elapsed in years since panel entry, rounded to the nearest integer for the purpose of the table (columns). A substantive number of observations occur in the off-diagonal cells, showing the varying time lags between observation points.

Separating age, cohort and period effects is an ongoing problem and area of research. The issue is that it is impossible to identify all three components as they are linear combinations of each other. To minimize any period effects, we used pre-pandemic data. Additionally, we restricted the age range to 25–70 to cover the adult lifespan and to ensure we have several different cohorts observed at equal ages (see Fig. 6, Appendix). Additionally, we control for the unemployment rate and the GDP growth rate, as the observation period overlaps with the 2008 economic crises, which have been shown to impact values (Vecchione, 2022; Sortheix & Schwartz, 2017).

All models were estimated according to Equation (1), where *y* refers to one of seven value measurements, *i* refers to the *i^th^* observation of respondent *j*. The $\beta_{0}$ parameter captures the population intercept, $\beta_{1}$ is the effect of one unit of time (year) increase since entry to the panel, in other words, the average intra-individual change in values over time. The $\beta_{2}$ captures differences in values between respondents of different ages at the time of entry to the panel, measuring age for the respondent *j* at the 1st observation. The $\beta_{3}$ captures differences in values between genders. The $\beta_{4}$ captures possible differences in the rate of intra-individual change between respondents of different ages. Similarly, $\beta_{5}$ captures the differences in the rate of intra-individual change between genders. The $\beta_{6}$ captures differences in the effect of gender across respondents of different ages. The $\beta_{7}$ and $\beta_{8}$ capture the effects of unemployment rate and GDP growth rate, respectively. Lastly the term $u_{0j}$ refers to the random intercept which captures unobserved differences between respondents and $u_{1j}time$ is the random slope of the variable time for each individual *j.*

(1) $$y_{\mathrm{ij}}=\beta_{\text{0}}+\beta_{1}{Time}_{ij}+\;\beta_{2}{Age}_{j}+\;\beta_{3}{Gender}_{j}{+\;\beta }_{\mathrm{cj}}{Cohort}_{j}+\beta_{4}{Time}_{ij}*{Age}_{j}\;+\beta_{5}{Time}_{ij}*{Gender}_{j}+\beta_{6}{Age}_{j}*{Gender}_{j}+\beta_{7}{Unemployment}_{ij}+\beta_{8}GDP\;Growth+u_{0j}+u_{1j}time$$

All multilevel model analyses were performed in Stata 15 using the ‘mixed’ command (StataCorp, 2017). We also estimate the intraclass correlation (ICC) of the empty model, meaning a model that only includes the constant and random intercept. The ICC shows the percentage variance between persons. Lastly, we also decompose the variance explained by the predictors in equation (1), the random intercepts and the random slopes using the r2mlm package [version 0.3.7] (Shaw et al., 2022) in R 4.3.2 (see Table 2).

## 3 Results

### 3.1 Intraclass correlation

The overall variance observed in ipsatised values can be decomposed into between and within-person variance using the intraclass correlation (ICC). The ICC shows what percentage of variance is between-persons. The ICCs shown in Table 2 range from 0.450 to 0.587. Therefore, the variance in the data can be almost equally divided in value differences between individuals and value change within individuals. The value with the lowest ICC is hedonism, indicating that hedonism changes most within individuals. On the other hand, conformity values have the highest ICC indicating the majority of variance is in differences between individuals.

**Table 2:** Intraclass Correlation Coefficient of Ipsatised and Standardized Value Scales.

	HE	CO	UN	BE	SD	ST	AC
Observations	34,129	34,129	34,129	34,129	34,129	34,129	34,129
Number of groups	10,860	10,860	10,860	10,860	10,860	10,860	10,860
ICC	0.450	0.587	0.534	0.485	0.539	0.508	0.521
S.E.	0.006	0.005	0.006	0.006	0.006	0.006	0.006
Upper bound	0.461	0.597	0.545	0.497	0.549	0.519	0.532
Lower bound	0.438	0.577	0.523	0.474	0.528	0.497	0.510

*Note*: Upper and lower bound correspond to the 95 % confidence interval. HE=hedonism. CO=conformity. UN=universalism. BE=benevolence. SD=self-direction. ST=Stimulation, AC=Achievement.

Variance explained by the multilevel models is shown in Table 3. The individual level predictors (fixed effects) and the individual slopes of time (random slope) explain a small proportion of the variance while the bulk of the explained variance is due to average differences between individuals (random intercept). These numbers indicate that the predictors, age, gender, cohort and time spent in the panel capture a limited proportion of the variation in values between and within persons.

**Table 3:** Explained Variance Attributable to the Predictors in Equation (1), Random Intercepts and Random Slopes.

Variance explained	CO	UN	BE	SD	ST	HE	SE
Fixed effect	0.015	0.057	0.031	0.004	0.043	0.038	0.031
Random intercept	0.570	0.481	0.455	0.533	0.467	0.412	0.493
Random slope	0.012	0.019	0.018	0.013	0.018	0.020	0.024
Total	0.596	0.557	0.505	0.550	0.528	0.470	0.548

*Note.* HE=hedonism. CO=conformity. UN=universalism. BE=benevolence. SD=self-direction. ST=Stimulation, AC=Achievement.

### 3.2 Mean-level changes

#### 3.2.1 Mean-level changes in personal values across age

**Fig. 2:**
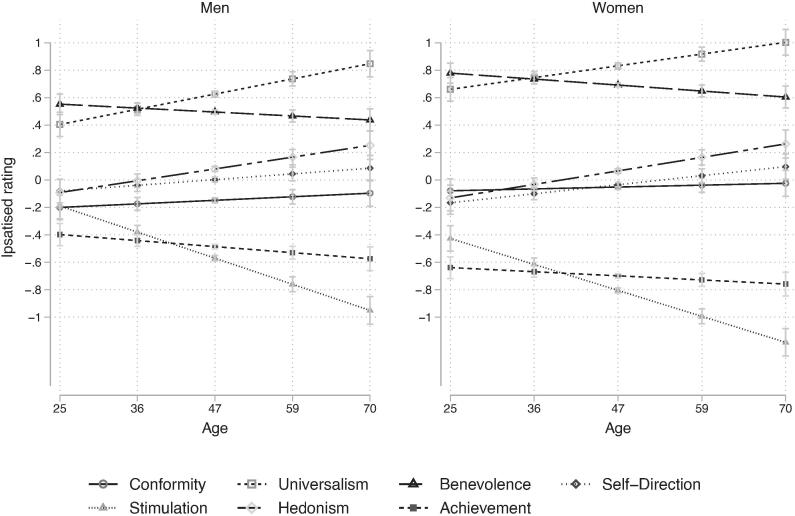
Average Values in Men and Women at Age of Panel Entry Note: Predicted preference for values across age at entry to the panel for men and women holding other covariates as observed. Using multilevel model as in equation (1) and ipsatised value measurements. X-axis five ages at panel entry (25, 36, 47, 59 and 70) corresponding to the minimum, 25th, 50th and 75th percentile and maximum age of panel entry observed in the data.

Fig. 2 shows mean-level differences in values across age at panel entry from multilevel models of ipsatised value measures (Appendix, Table 8). To judge whether differences shown in Fig. 2 are significant, we estimated the marginal effects of age at panel entry at one standard deviation above (39 years old) and below (59 years old) the mean age at panel entry. Then we estimated whether these were significantly different using a Bonferroni adjustment for the p-values. The results show that older respondents value universalism (*β* = 0.283, *p* < 0.001), self-direction (*β* = 0.171, *p* < 0.011) and hedonism (*β* = 0.247, *p* < 0.001) more than younger respondents while stimulation values are less important (*β* = −0.520, *p* < 0.001) for older respondents compared to younger respondents. Additionally, neither conformity (*β* = 0.058, *p* = 0.391), achievement (*β* = −0.118, *p* = 0.074), nor benevolence (*β* = −0.127, *p* = 0.051) change significantly in importance across age groups.

Age differences must be interpreted with caution as we do not observe all cohorts across the full age range of 25–70, thus, it is possible that cohort and age effects are confounded, for example, if age trends differ across cohorts. For example, today’s younger respondents may differ in their conformity values in their late adulthood compared to today’s late adulthood respondents due to e.g. later age of marriage and child-rearing.

***Cohort effects*.** We observe lower preference for hedonism values for older cohorts compared to younger cohorts (see Table 8 and Fig. 7 in Appendix). These cohort effects are large and come out clearly for cohorts where we have the most data, meaning observations with overlapping age at entry. Other cohort effects are less evident and maybe due to the fact we do not have data on comparable ages for the oldest and youngest cohorts. These cohort effects include a small decrease in the importance of stimulation, benevolence and conformity values for 1978–1995 cohort. Additionally, the 1969–1973 cohort shows lower achievement values than older cohorts.

**Fig. 3:**
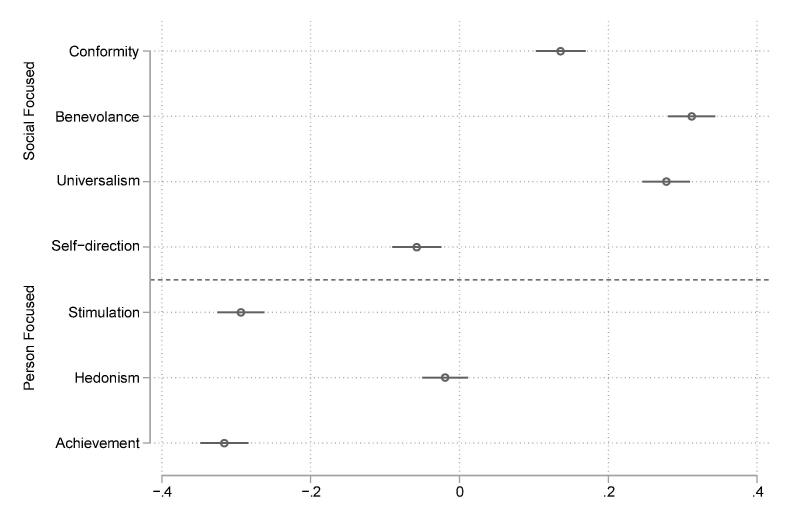
Average Differences in Value Scales (Ipsatised and Standardized) Between Genders. Note: Positive effects indicate women attribute a higher relative importance to the value than men.

***Gendered value differences between age*.** We estimated the average marginal effect of gender on each value using the standardized estimates of the ipsatised value measures, see Fig. 3. On average, the differences were greatest in values of achievement (*β* = −0.316, *p* < 0.001), benevolence (*β* = 0.312, *p* < 0.001), stimulation (*β* = −0.294, *p* < 0.001) and universalism (*β* = 0.312, *p* < 0.001) and much smaller for self-direction (*β* = −0.057, *p* < 0.001) and conformity values (*β* = 0.136, *p* < 0.001) while not significant for hedonism (*β* = -0.019, *p* < 0.221). Note that the largest differences in value priorities were in those values that were rated most important (universalism and benevolence) and least important (achievement and stimulation) by both genders. Showing that the ranking of values was similar but the differences in the relative importance and ratings were substantive.

Gender differences in value ratings differ across age groups, as shown by the interaction effects between age and gender (Appendix, Table 8). We report the gender differences between genders at one standard deviation above and below the mean using Bonferroni's adjustment to compute the *p*-value. The differences in the importance of hedonism, achievement, conformity, benevolence, and stimulation do not change significantly with age. Gender differences in the importance of universalism (*β* = −0.073, *p* = 0.046) and self-direction (*β* = 0.076, *p* = 0.048) increase with age. Women find universalism more important than men on average, and the gap increases in older age groups. Men find self-direction more important than women on average and the gap increases in older age groups.

### 3.3 Rate of intra-individual change

***Within-person change*.** We estimate and report marginal effects of time which are the average change in values (ipsatised and standardised) associated with a one year increase in time. The regression results are shown in Appendix Table 8. Hedonism (*β* = 0.008, *p* < 0.001), universalism (*β* = 0.021, *p* < 0.001), stimulation (*β* = −0.015, *p* < 0.001) and achievement (*β* = −0.015, *p* < 0.001) have significant rates of change on average. Conformity (*β* = 0.02, *p* = 0.252), benevolence (*β* = −0.001, *p* < 0.581) and self-direction (*β* = −0.001, *p* < 0.716) values do not have a significant intra-individual change.

**Fig. 4:**
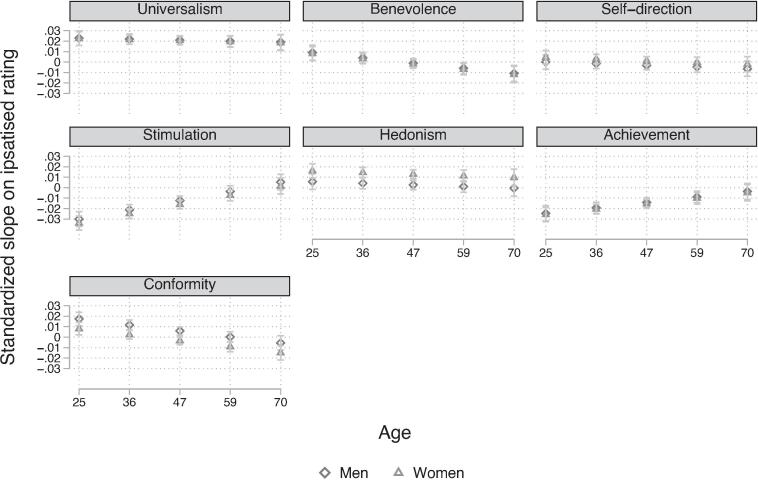
Standardized Rates of Change of Ipsatised Value Measures Across Age *at Panel Entry Note*: Estimated average intra-individual rate of change from multilevel model (equation (1) using standardized and ipsatized value measures from the LISS panel.

**Figure 6:**
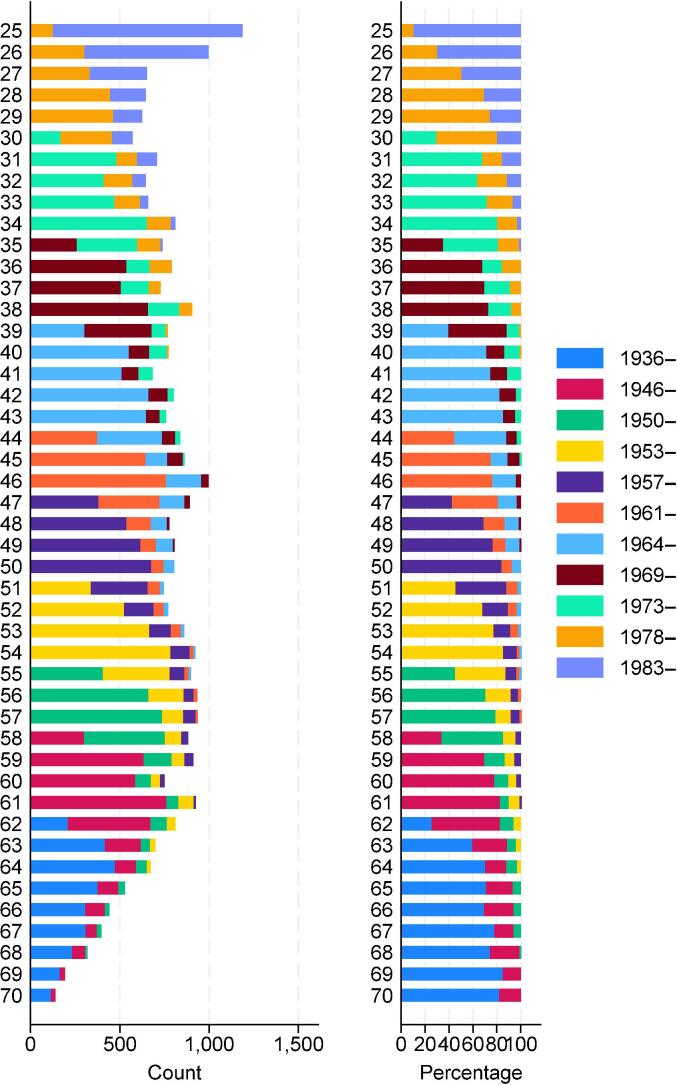
The Number And Percentage Of Observations By Their Age At Panel Entry And Cohort

**Figure 7:**
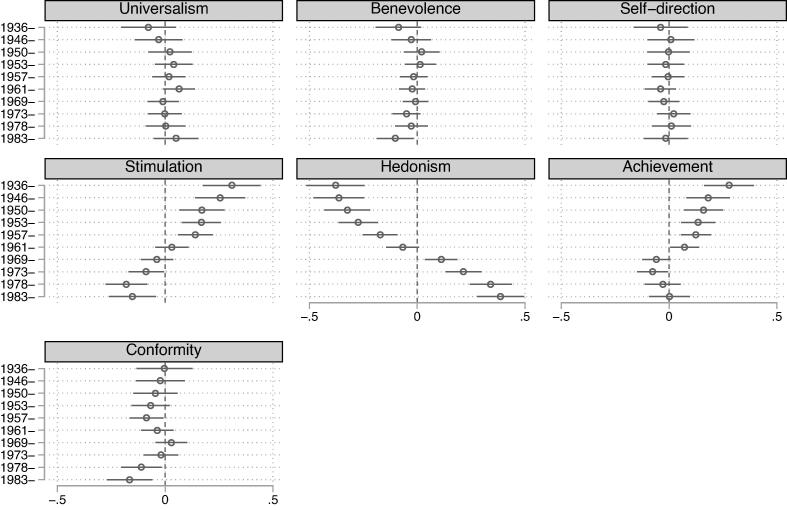
Differences In Value Preferences Between Cohorts With 1964-1968 As Reference.

Additionally, the rate of change has significant interaction effects with the age at panel entry, as shown in Fig. 4 (also see Appendix, Table 8). According to estimated marginal effects, the rates of change do not significantly increase nor decrease between one standard deviation below (39) or above (59) the mean age for universalism (*β* = −0.002, *p* = 1.000), hedonism (*β* = −0.003, *p* = 1.000) and self-direction (*β* = −0.003, *p* = 1.000). The rate of change in benevolence (*β* = −0.011, *p* = 0.007) and conformity (*β* = −0.013, *p* < 0.001) differs significantly with age. The rate of change shifts from positive in young respondents to negative in older respondents. The rate of change in stimulation (*β* = 0.019, *p* < 0.001) and achievement (*β* = 0.011, *p* = 0.004) values show the opposite dynamic, younger respondents have a negative rate of change which increases significantly up to older populations, when the rate of change is nullified.

Lastly, the differences in the rates of change between men and women are compared at age 36 and age 59 (one standard deviation below and above the mean age). Gender differences in the rate of change are different at age 36 and 59 for hedonism (*β* = −0.010, *p* = 0.004) and conformity (*β* = 0.009, *p* < 0.002). Gender differences in the rate of change are equal and not significant at age 36 and 59 for universalism (*β* = 0.000, *p* = 1.000), benevolence (*β* = 0.001, *p* = 1.000*),* self-direction, universalism (*β* = −0.040, *p* = 0.666), stimulation (*β* = 0.004, *p* = 1.000) and security (*β* = 0.001, *p* = 1.000). Thus, rates of change are similar for both genders across different ages at panel entry for all values except hedonism and conformity values.

## 4 Discussion

The present research examined the value development of a representative sample of Dutch adults from age 25–70 over an 11-year period from 2008 to 2019. We find clear differences in value preferences between respondents of different ages and genders as well as intra-individual change over time. In fact, about half of all variation in the sample is within-person variation for the majority of values. We interpret the results using the neo-analytic model of personality development and observe trends in values that fit the maturation principle.

### 4.1 Many roads to the same destination

Differences between ages are partly supportive of the maturity principle which states that older respondents are more oriented towards society and functioning within groups. We found that older respondents value universalism, self-direction and hedonism more while they value stimulation less than younger respondents. This indicates that older respondents generally value their ability to think and act as they see fit (self-direction), to ensure the inclusion and respect to all members of society (universalism) and to be comfortable (hedonism) and are much less stimulation seeking. Thus, values are not simply more social-focused for older respondents but do seem to be conducive to engaging with and contributing to Dutch society.

We observed values changing within individuals across all ages, contrary to the ‘formative years’ hypothesis and more in line with NSM's plasticity principle. We find the largest rates of change in early adulthood in line with other studies (Leijen et al., 2022, Smallenbroek et al., 2023) and also find smaller rates of change in middle adulthood, as commonly assumed. The rates of change do indicate there is an early developmental period where respondents are increasingly social focused in values (universalism, benevolence, conformity) and decrease the importance of personal focused values (achievement and stimulation). These rates of intra-individual change again provide evidence for the maturity principle as respondents become more social focused at the age where Dutch individuals typically start to form partnerships, families and may have to take care of elderly family members.

A novel finding, however, is that older adults also tend to decrease the relative importance of benevolence and conformity values which are generally assumed to be more important in later life. Additionally, stimulation and achievement values stabilize, instead of decreasing. Moreover, the only value that is consistently increasing over the lifespan for both genders is universalism. Women of all ages also report a positive rate of change for the relative importance of hedonism. Overall, the rates of change indicate some tendency towards growth values, in contrast to the maturation principle. These findings could also be interpreted as evidence for NSM’s plasticity principle which states that values can change in response to the environment at any age.

### 4.2 Gender-specific value development

We observe gender differences across all values in line with previous research (Borg, 2019, Schwartz and Rubel, 2005, Schwartz and Rubel-Lifschitz, 2009, Vilar et al., 2020). We find that universalism, benevolence and conformity values were more important for women whereas self-direction, stimulation and achievement values were more important for men. However, these gender differences do not impact the ranking of values; men and women rank values similarly. Gender differences in universalism and self-direction increase with age. This indicates gender differences in values are mainly formed early in life and sustained through adulthood.

Comparing value’s importance across age groups and gender, we observe changes in the value priorities or ranking of values. It seems that the priorities of men and women converge in older age groups, suggesting a gendered value-development that could be interpreted and investigated further with the social-investment principle of NSM. There is also considerable stability in the value priorities across age groups, with largely three sets of values. Benevolence and universalism values are highly ranked, hedonism, conformity and self-direction are middle ranking while achievement and stimulation compete at the lowest rankings. We note that mean-level changes between age groups in the panel could also be due to an interaction between gender and cohort differences or changes in the lifespan development between cohorts. Further research is needed to understand how the value priority structures of men and women develop.

We also found that the rate of intra-individual change differed between genders in the values of conformity and hedonism. These results indicate that gender differences in values continue to develop in adulthood to a small extend rather than being exclusively formed earlier in life, when personality traits and values changes have been observed (Caspi et al., 2005, Vecchione et al., 2016, Schwartz, et al., 2019). Given the highly gendered structure of society and social interaction, it is not surprising to find gender differences in the importance and rates of change for several values in adulthood. Especially considering that the neo-socioanalytic model puts identity as a fulcrum of assessment.

Despite the many age, gender and time effects, the explanatory variables explain little of the variation found in the data. This indicates that gender differences in values in western societies may be sustained by the wide variety of gender differences in labor market participation and timing of life events such as the age at which partnerships are formed and the age at which couples have their first child (Billari & Liefbroer, 2010). Future research could investigate whether gender differences in values reflect these specific lifespan changes in identity and social roles within a country's institutional arrangements and culture.

### 4.3 Cohort changes in values

We observed few cohort differences contrary to well-established claims by political scientists (Inglehart, 1977), who argued that economic growth encourages more self-focused and less social-focused values in cohorts born after the world wars as compared to those born before and during. The only evidence we do find is that cohorts born during difficult economic times (before the 60 s) have lower hedonism values. We found that the unemployment rate has a negative effect on the relative importance of universalism while the GDP growth rate has a positive effect on benevolence and achievement values and a negative impact on stimulation. Thus, the economic effect on values is not simply a matter of more person/social focused values. Additionally, we do find the unemployment and GDP growth rates have a negative and positive impact, respectively, on all value *ratings*. This is an interesting finding pertinent to the measurement debate in personal values. Theoretically, the relative importance of values is what matters, but others argue that the ratings are also informative (Rudnev, 2021, Parks-Leduc et al., 2015).

### 4.4 Similarities and differences with Leijen et al. (2022) study

Despite the differences in methods, data, measurement and variables included in the model, we find similar results to Leijen et al. (2022). These are reassuring findings in the context of a replication crisis (e.g., Shrout & Rodgers, 2018). We find similar differences between age groups in universalism, benevolence, conformity, self-direction, hedonism, and stimulation. The two models attribute age differences to different parameters. In Leijen et al. (2022) age differences are attributed to cohorts whereas the results of this paper attribute these to age. The only value where the two models corroborate each other is in the cohort differences in hedonism. The two sets of results conflict in terms of age differences in achievement.

In terms of the rates of change, the two models corroborate each other in stimulation, benevolence, achievement (named power in Leijen et al., 2022) and conformity values. In terms of self-direction values, we find no change whereas Leijen et al. (2022) find a positive slope for younger respondents. Leijen et al. (2022) also find younger respondents increase universalism values, while we find an increase in universalism values for respondents of all ages. Lastly, we find respondents increase the valuation of hedonism slightly but consistently across ages while Leijen et al. (2022) do not find this effect statistically significant (possibly due to sample size). We both find gender differences in rates of change in hedonism and conformity.

In short, the results from the two studies mostly corroborate each other, giving further confidence in the results as two separate teams of researchers using different methods, samples and measurements come to similar conclusions. However, our model would ascribe differences in values between age groups mostly to age whereas Leijen et al. (2022) model ascribes these to cohort.

### 4.5 Limitations and future research directions

The advantage of the present data structure and analysis method is that we were able retain a high number of observations and time points while respecting the duration between each observation. The disadvantage, however, is that the calendar year of panel entry varies across respondents, which may induce some uncertainty or bias to cohort or age effects due to period effects. For example, the many wars European countries were involved in and the terrorist attacks that targeted European countries in these years may have impacted value development. However, we were able to control for the economic conditions.

Notably, the present research was carried out in the Netherlands, a country with a very high standard of living (UNDP, 2024) in addition to being prototypically culturally Western with a very high score on individualism and an orientation toward self-determination (e.g., Witte, Stanciu, & Boehnke, 2020). The findings from this context may not generalize to other contexts.

We have 11 years of observation which is a substantive improvement over most previous studies that use three-year longitudinal panel data. We observed changes that take a long time to occur and thus demonstrate the value of long-term longitudinal panel data. However, we present evidence from age 25 to 70 which overshoots the 11-year observation period. Without observing the whole lifespan of an individual, we cannot be certain whether the changes in values are due to period, cohort or lifespan effects. Nevertheless, we have several cohorts covering the same age range and control for cohort effects. Thus, within certain age ranges and over observed cohorts we can be more certain of the estimates presented in this paper.

We examined the age period 25–70 to contribute to the literature on value development in adulthood, as there were clear theoretical expectations of long term trends but little empirical evidence. We opted to exclude teenagerhood and old age as these periods are markedly different from adulthood. Teenagerhood is a period of rapid psychological development when values have high plasticity and match identity formation processes (Cieciuch et al., 2016; Erikson, 1997). Meanwhile, findings in the literature on older people are scarce. It is likely that values also change throughout old age as personality traits retain plasticity (Graham et al., 2020, Roberts and Nickel, 2017). However, value development at old age demands an examination that takes into account the impact of preparing for old age and death (Brandtstädter et al., 2010), data quality due to older people being a hard to reach population (Kammer, Falk, Herzog, & Fuchs, 2019), and, finally, a comparative study of the old (65+) and old old (80+) people with their unique challenges and opportunities due to advances in the health and technology sectors (Baltes & Smith, 2003). Integrating these considerations into a study of value development remains a challenging task beyond the scope of this paper.

## 5 Conclusion

Few studies have empirically investigated value development over the life span. Research on this topic has been hampered by a lack of longitudinal data and a lack of theoretical perspectives emphasizing change over the lifespan. As a result, most studies on value development use cross-sectional data and assume age differences reflect changes within the lifespan or focus on the early lifespan with shorter longitudinal datasets (Dobewall et al., 2017, Gouveia et al., 2015, Milfont et al., 2016; Vecchione et al., 2016, Vecchione et al., 2019, Vilar et al., 2020). However, evidence from longitudinal studies in personal values and personality traits do show continuous personality change and have generated advances in theory (Smallenbroek et al., 2023, Bardi and Goodwin, 2011, Leijen et al., 2022, Milfont et al., 2016, Roberts and Nickel, 2017). This paper seeks to add to this literature by providing a comprehensive empirical study of value development over the life span using longitudinal data spanning 11 years.

In this paper we used the Neo-Socioanalyitcal Model of personality to examine value development over 11 years in a nationally representative sample of Dutch respondents. We fitted multilevel growth models which included a random slope for intra-individual change over time, and fixed effects to capture differences between individuals, of different ages at panel entry, cohort and gender. We found evidence for continuous change over the life span. We find individuals who entered the panel before their 50s shifting to a more social-focused value profile after which values seem to stabilize. Only two values change in older respondents, namely universalism (men and women) and hedonism (women). This pattern of value change can be interpreted as fitting the maturation principle as a development towards adjusting to societal demands in mid-adulthood and towards growth values in late adulthood. Additionally, we find limited gender differences in the rates of change across age groups. Thus, men and women seem to change values at the same rate and in the same direction. However, gender differences in values seem to form before the age of 25 and remain largely stable throughout adulthood.

This paper adds to the existent ambiguity in the literature in view of exact timing and trajectories of value changes. The prevailing consensus in the values literature is that social-focused values increase, and personal-focused values decrease with age. However, this no longer seems specific enough. Milfont et al., 2016, Leijen et al., 2022, Smallenbroek et al., 2023 and the present results show that value development is non-linear and the rate of change dynamics and mean-level changes across age show value specific periods of change and stability. These results also imply that cross-sectional studies confound age and cohort effects when examining the effect of age on values. Lastly, the models presented leave a substantial portion of variance unexplained while the variation in random slopes indicates some individuals change twice as fast than average and others do not change at all. These estimates show that there is a lot more to unpack and understand about why some people's values change while others do not.

## CRediT authorship contribution statement

**Oscar Smallenbroek:** Writing – review & editing, Writing – original draft, Methodology, Formal analysis, Conceptualization. **Adrian Stanciu:** Writing – review & editing, Writing – original draft, Visualization, Methodology, Conceptualization.

## Declaration of competing interest

The authors declare that they have no known competing financial interests or personal relationships that could have appeared to influence the work reported in this paper.

## Figures and Tables

**Table 4:** Model fit indices for multi-group factor analysis.

Factor	AIC	BIC	χ^2^			RMSEA [*90 % CI]*			CFI	TLF	SRMSR	CoD	Groups (Survey waves)
			*Coef.*	*df*	*p-value*	*Coef.*							
Conservation	311683.63	312012.72	231.61	60	0.00	0.03	[0.03	0.03]	1.00	1.00	0.03	0.85	11
Universalism	280655.22	280984.28	252.33	60	0.00	0.03	[0.03	0.04]	0.99	1.00	0.05	0.80	11
Benevolence	428172.72	428721.19	2326.67	155	0.00	0.07	[0.06	0.07]	0.97	0.98	0.08	0.87	11
Achievement	325885.28	326214.38	429.39	60	0.00	0.04	[0.04	0.05]	0.98	0.99	0.04	0.77	11

*Note*. Coef. = coefficient. AIC=Akaike Information Criterion. BIC=Bayesian Information Criterion. RMSEA=Root Mean Squared Error of Apporximation (*90 % CI l* = 90 % CI lower bound; *90 % CI u* = 90 % CI upper bound). CFI=Comparative Fit Index. TL=Tucker-Lewis Fit Index. SRMSR=Standardized Root Mean Squared Residual. CoD=Coefficient of Determination. Group = Number of Groups.4

**Table 5:** Model parameter estimates for multi-group CFA of person focused values of Rokeach value survey items in the LISS data.

	Universalism		Benevolence		Conformity		Achievement	
Parameter	Item	Coef.	Item	Coef.	Item	Coef.	Item	Coef.
Loading	a world at peace	0.845	sincere and truthful	0.560	clean	1.136	intellectual	0.741
		(0.008)		(0.004)		(0.007)		(0.008)
Loading	freedom	0.680	forgiving	0.762	self-controlled	1.031	a sense of accomplishment	1.039
		(0.005)		(0.006)		(0.006)		(0.008)
Loading	equality	0.882	open-minded	0.779	obedient	0.884	social recognition	0.969
		(0.006)		(0.006)		(0.008)		(0.008)
Loading			helpful	0.828				
				(0.005)				
Loading			loving	0.824				
				(0.005)				
Intercept	a world at peace	5.731	sincere and truthful	6.546	clean	5.218	intellectual	4.817
		(0.007)		(0.005)		(0.007)		(0.007)
Intercept	freedom	6.442	forgiving	5.580	self-controlled	5.291	a sense of accomplishment	4.863
		(0.005)		(0.006)		(0.007)		(0.007)
Intercept	equality	6.116	open-minded	5.674	obedient	4.738	social recognition	4.614
		(0.006)		(0.006)		(0.008)		(0.008)
Intercept			helpful	5.966				
				(0.005)				
Intercept			loving	6.113				
				(0.005)				
Mean Latent		0.000		0.000		0.000		0.000
		(0.000)		(0.000)		(0.000)		(0.000)
Variance Latent		1.000		1.000		1.000		1.000
		(0.000)		(0.000)		(0.000)		(0.000)
Error Variance	a world at peace	1.028	sincere and truthful	0.358	clean	0.429	intellectual	1.174
		(0.023)		(0.008)		(0.017)		(0.025)
Error Variance	freedom	0.306	forgiving	0.751	self-controlled	0.495	a sense of accomplishment	0.548
		(0.009)		(0.016)		(0.016)		(0.021)
Error Variance	equality	0.395	open-minded	0.690	obedient	1.257	social recognition	0.968
		(0.013)		(0.015)		(0.026)		(0.025)
Error Variance			helpful	0.313				
				(0.008)				
Error Variance			loving	0.283				
				(0.008)				
N		34,129		34,129		34,129		34,129

**Table 6:** Sample Selection LISS data waves 2008–2019.

Criterion	*N*
Sample who responded to RVS	44,854
Age 25–70	34,484
Missing Value Measures	34,177
Inconsistent change Gender	34,169
Inconsistent change of Birth year	34,129

Note: N is the number of cases remaining after applying the criterion in the left column.

**Figure 5:**
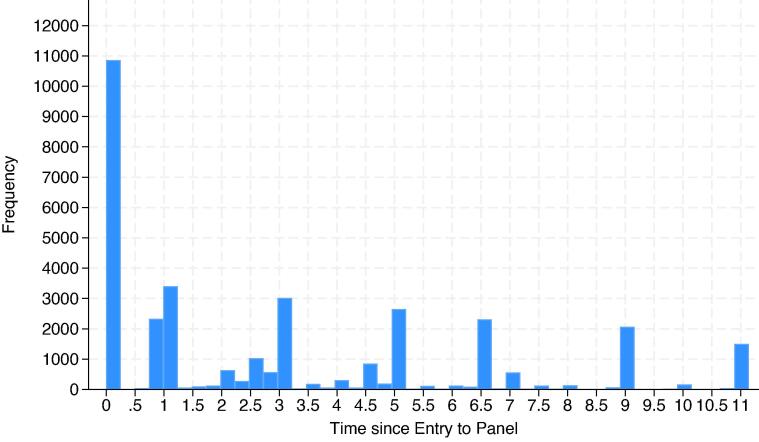
The Frequency of Observations At Each Time Point At Which Respondents Were Observed Since Panel Entry.

**Table 7:** Number of observations for each nth observation point and corresponding time since first observation.

n_th_	0	1	2	3	4	5	6	7	8	9	10	11	*Total*
1	12,233	0	0	0	0	0	0	0	0	0	0	0	12,233
2	0	6,472	1,674	476	91	18	11	6	0	1	1	1	8,751
3	0	0	468	3,914	955	310	90	38	10	14	1	1	5,801
4	0	0	0	2	332	3,028	456	179	41	54	17	14	4,123
5	0	0	0	0	0	3	2,023	745	153	286	44	37	3,291
6	0	0	0	0	0	0	1	4	3	1,940	139	197	2,284
7	0	0	0	0	0	0	0	0	0	4	3	1,401	1,408
8	0	0	0	0	0	0	0	0	0	0	0	3	3
*Total*	12,233	6,472	2,142	4,392	1,378	3,359	2,581	972	207	2,299	205	97	37,894

*Note. Coloum 1:* n_th_ = Number of times a respondent was observed in the panel. Columns 2–12 specify the time in years (rounded) since panel entry, with 0 equating panel entry.

**Table 8:** Regression table of multilevel models of standardized ipsatised value measures.

	Universalism	Benevolence	Self-direction	Stimulation	Hedonism	Achievement	Conformity
Time	0.021***	−0.000	−0.003	−0.013***	0.003	−0.015***	0.007***
	(0.002)	(0.002)	(0.002)	(0.002)	(0.002)	(0.002)	(0.002)
Age at panel entry	0.165***	−0.033	0.070*	−0.290***	0.120***	−0.088**	0.058
	(0.033)	(0.034)	(0.035)	(0.033)	(0.033)	(0.034)	(0.035)
Gender (ref. Men)	0.279***	0.315***	−0.071***	−0.282***	−0.050**	−0.312***	0.164***
	(0.017)	(0.017)	(0.018)	(0.017)	(0.017)	(0.017)	(0.018)
Cohort (Ref. 1964–1968)	0.018	0.071	−0.101	0.277***	−0.462***	0.189**	0.063
1937–1945	(0.069)	(0.069)	(0.072)	(0.069)	(0.068)	(0.070)	(0.073)
	0.057	0.120*	−0.030	0.215***	−0.434***	0.112	0.019
1946–1949	(0.060)	(0.060)	(0.063)	(0.060)	(0.059)	(0.061)	(0.064)
	0.111*	0.179**	−0.057	0.139*	−0.378***	0.072	−0.006
1950–1952	(0.055)	(0.055)	(0.057)	(0.055)	(0.054)	(0.056)	(0.058)
	0.106*	0.124**	−0.035	0.132**	−0.326***	0.080	−0.031
1953–1956	(0.047)	(0.047)	(0.049)	(0.047)	(0.046)	(0.048)	(0.050)
	0.081	0.086*	−0.015	0.107**	−0.206***	0.054	−0.076
1957–1960	(0.042)	(0.041)	(0.043)	(0.041)	(0.040)	(0.042)	(0.044)
	0.081*	0.013	−0.031	0.047	−0.097*	0.041	−0.044
1961–1963	(0.040)	(0.039)	(0.041)	(0.039)	(0.038)	(0.040)	(0.042)
	0.279***	0.315***	−0.071***	−0.282***	−0.050**	−0.312***	0.164***
1969–1972	−0.025	−0.050	−0.013	−0.032	0.154***	−0.082*	0.022
	(0.038)	(0.038)	(0.040)	(0.038)	(0.037)	(0.039)	(0.040)
1973–1977	−0.019	−0.125**	0.055	−0.084*	0.253***	−0.082	−0.044
	(0.041)	(0.041)	(0.043)	(0.041)	(0.040)	(0.042)	(0.044)
1978–82	−0.051	−0.159**	0.031	−0.150**	0.407***	0.013	−0.140**
	(0.049)	(0.049)	(0.051)	(0.048)	(0.048)	(0.049)	(0.051)
1983–1994	−0.025	−0.307***	0.034	−0.135*	0.489***	0.086	−0.221***
	(0.055)	(0.055)	(0.057)	(0.055)	(0.054)	(0.056)	(0.058)
Time x Age at panel entry	−0.001	−0.005**	−0.002	0.010***	−0.002	0.006***	−0.006***
	(0.002)	(0.002)	(0.002)	(0.002)	(0.002)	(0.002)	(0.001)
Gender x Time	−0.000	−0.001	0.004	−0.004	0.010***	−0.001	−0.009***
	(0.003)	(0.003)	(0.003)	(0.003)	(0.003)	(0.003)	(0.003)
Gender x Age at panel entry	−0.037*	−0.026	0.038*	0.001	0.017	0.023	−0.019
	(0.015)	(0.015)	(0.016)	(0.015)	(0.015)	(0.015)	(0.016)
Unemployment rate (%)	−0.011***	0.003	0.006	0.002	−0.005	0.005	0.001
	(0.003)	(0.003)	(0.003)	(0.003)	(0.003)	(0.003)	(0.003)
GDP Growth (%)	0.010	0.023**	−0.011	−0.015*	−0.009	−0.010	0.017*
	(0.007)	(0.007)	(0.007)	(0.007)	(0.007)	(0.007)	(0.007)
Constant	−0.188***	−0.208***	0.033	0.169***	0.089**	0.157***	−0.091**
	(0.032)	(0.033)	(0.033)	(0.033)	(0.033)	(0.033)	(0.033)
S.D.(Slope Time)	0.042***	0.041***	0.035***	0.041***	0.043***	0.047***	0.033***
	(0.002)	(0.003)	(0.003)	(0.003)	(0.003)	(0.002)	(0.003)
S.D.(Intercept)	0.705***	0.687***	0.753***	0.691***	0.666***	0.716***	0.782***
	(0.008)	(0.008)	(0.008)	(0.008)	(0.008)	(0.008)	(0.008)
r(I,S)	−0.200***	−0.208***	−0.300***	−0.174***	−0.321***	−0.187***	−0.337***
	(0.039)	(0.041)	(0.043)	(0.041)	(0.039)	(0.037)	(0.043)
S.D.(Error)	0.665***	0.702***	0.671***	0.685***	0.726***	0.674***	0.637***
	(0.003)	(0.004)	(0.003)	(0.003)	(0.004)	(0.003)	(0.003)
BIC	84,904	87,318	85,977	86,153	88,450	86,118	83,845
AIC	84710.03	87123.6	85782.8	85959.09	88255.51	85924.3	83650.9
N	34,129	34,129	34,129	34,129	34,129	34,129	34,129

*Note*. *r*(I,S) = correlation between intercept and slope. *p < 0.05, ** p < 0.01, *** p < 0.001. Standard errors in parentheses.

**Table 9:** Regression table of multilevel models of ipsatised value measures.

	Universalism	Benevolence	Self-direction	Stimulation	Hedonism	Achievement	Conformity
Time	0.016***	−0.000	−0.002	−0.011***	−0.010***	0.005***	0.002
	(0.002)	(0.001)	(0.001)	(0.002)	(0.002)	(0.001)	(0.002)
Age at panel entry	0.124***	−0.021	0.049*	−0.232***	−0.060**	0.042	0.099***
	(0.025)	(0.021)	(0.025)	(0.027)	(0.023)	(0.025)	(0.027)
Gender (ref. Men)	0.210***	0.200***	−0.050***	−0.226***	−0.212***	0.118***	−0.041**
	(0.013)	(0.011)	(0.013)	(0.014)	(0.012)	(0.013)	(0.014)
Cohort (Ref. 1964–1968) 1937–1945	0.014	0.045	−0.071	0.221***	0.128**	0.046	−0.380***
	(0.052)	(0.044)	(0.051)	(0.055)	(0.048)	(0.053)	(0.056)
1946–1949	0.043	0.076*	−0.021	0.172***	0.076	0.014	−0.357***
	(0.045)	(0.038)	(0.044)	(0.048)	(0.042)	(0.046)	(0.049)
1950–1952	0.083*	0.114**	−0.040	0.111*	0.049	−0.004	−0.311***
	(0.041)	(0.035)	(0.040)	(0.044)	(0.038)	(0.042)	(0.044)
1953–1956	0.080*	0.079**	−0.025	0.106**	0.055	−0.023	−0.268***
	(0.035)	(0.030)	(0.034)	(0.038)	(0.032)	(0.036)	(0.038)
1957–1960	0.061	0.055*	−0.010	0.085**	0.037	−0.055	−0.170***
	(0.031)	(0.026)	(0.030)	(0.033)	(0.029)	(0.032)	(0.033)
1961–1963	0.061*	0.008	−0.022	0.038	0.028	−0.032	−0.080*
	(0.030)	(0.025)	(0.029)	(0.031)	(0.027)	(0.030)	(0.032)
1969–1972	−0.019	−0.032	−0.009	−0.025	−0.056*	0.016	0.126***
	(0.029)	(0.024)	(0.028)	(0.030)	(0.026)	(0.029)	(0.030)
1973–1977	−0.014	−0.079**	0.039	−0.067*	−0.055	−0.032	0.208***
	(0.031)	(0.026)	(0.030)	(0.033)	(0.029)	(0.032)	(0.033)
1978–82	−0.038	−0.101**	0.022	−0.120**	0.009	−0.101**	0.335***
	(0.037)	(0.031)	(0.036)	(0.039)	(0.033)	(0.037)	(0.039)
1983–1994	−0.019	−0.195***	0.024	−0.108*	0.059	−0.160***	0.403***
	(0.041)	(0.035)	(0.040)	(0.044)	(0.038)	(0.042)	(0.045)
Time x Age at panel entry	−0.001	−0.003**	−0.001	0.008***	0.004***	−0.005***	−0.001
	(0.001)	(0.001)	(0.001)	(0.001)	(0.001)	(0.001)	(0.001)
Gender x Time	−0.000	−0.001	0.003	−0.003	−0.001	−0.007***	0.008***
	(0.002)	(0.002)	(0.002)	(0.002)	(0.002)	(0.002)	(0.003)
Gender x Age at panel entry	−0.027*	−0.016	0.027*	0.001	0.015	−0.013	0.014
	(0.011)	(0.010)	(0.011)	(0.012)	(0.010)	(0.012)	(0.012)
Unemployment rate (%)	−0.008***	0.002	0.004	0.001	0.003	0.001	−0.004
	(0.002)	(0.002)	(0.002)	(0.002)	(0.002)	(0.002)	(0.003)
GDP Growth (%)	0.007	0.015**	−0.008	−0.012*	−0.006	0.012*	−0.007
	(0.005)	(0.005)	(0.005)	(0.006)	(0.005)	(0.005)	(0.006)
Constant	0.588***	0.478***	−0.007	−0.552***	−0.494***	−0.151***	0.138***
	(0.024)	(0.021)	(0.024)	(0.026)	(0.022)	(0.024)	(0.027)
S.D.(Slope Time)	0.031***	0.026***	0.024***	0.033***	0.032***	0.024***	0.035***
	(0.002)	(0.002)	(0.002)	(0.002)	(0.002)	(0.002)	(0.002)
S.D.(Intercept)	0.530***	0.436***	0.528***	0.552***	0.486***	0.564***	0.548***
	(0.006)	(0.005)	(0.006)	(0.006)	(0.005)	(0.006)	(0.007)
r(I,S)	−0.200***	−0.208***	−0.300***	−0.174***	−0.187***	−0.337***	−0.321***
	(0.039)	(0.041)	(0.043)	(0.041)	(0.037)	(0.043)	(0.039)
S.D.(Error)	0.500***	0.446***	0.471***	0.548***	0.458***	0.459***	0.598***
	(0.003)	(0.002)	(0.002)	(0.003)	(0.002)	(0.002)	(0.003)
BIC	65,462	56,342	61,801	70,892	59,678	61,576	75,210
AIC	65268.4	56148.35	61607.4	70698.32	59484.13	61382.41	75015.9
N	34,129	34,129	34,129	34,129	34,129	34,129	34,129

*Note*. *r*(I,S) = correlation between intercept and slope. *p < 0.05, ** p < 0.01, *** p < 0.001. Standard errors in parentheses.

**Table 10:** Regression table of multilevel models using ratings of value.

	Universalism	Benevolence	Self-direction	Stimulation	Hedonism	Achievement	Conformity
Time	−0.001	−0.017***	−0.019***	−0.027***	−0.015***	−0.027***	−0.012***
	(0.002)	(0.002)	(0.002)	(0.002)	(0.003)	(0.002)	(0.002)
Age at panel entry	0.188***	0.044	0.118**	−0.163***	0.164***	0.009	0.111**
	(0.030)	(0.027)	(0.036)	(0.040)	(0.039)	(0.037)	(0.037)
Gender (ref. Men)	0.284***	0.273***	0.023	−0.153***	0.032	−0.139***	0.191***
	(0.016)	(0.014)	(0.018)	(0.020)	(0.020)	(0.019)	(0.019)
Cohort (Ref. 1964–1968)							
1937–1945	0.098	0.131*	0.007	0.299***	−0.296***	0.206**	0.125
	(0.063)	(0.056)	(0.074)	(0.084)	(0.080)	(0.077)	(0.077)
1946–1949	0.096	0.131**	0.027	0.220**	−0.304***	0.122	0.063
	(0.055)	(0.048)	(0.065)	(0.073)	(0.070)	(0.068)	(0.067)
1950–1952	0.080	0.112*	−0.049	0.102	−0.312***	0.039	−0.008
	(0.050)	(0.044)	(0.059)	(0.067)	(0.064)	(0.062)	(0.062)
1953–1956	0.124**	0.123**	0.017	0.146*	−0.224***	0.096	0.022
	(0.042)	(0.038)	(0.051)	(0.057)	(0.054)	(0.053)	(0.053)
1957–1960	0.097**	0.091**	0.026	0.122*	−0.133**	0.072	−0.017
	(0.037)	(0.033)	(0.045)	(0.051)	(0.048)	(0.047)	(0.047)
1961–1963	0.070*	0.018	−0.015	0.045	−0.072	0.034	−0.024
	(0.036)	(0.031)	(0.043)	(0.048)	(0.046)	(0.045)	(0.044)
1969–1972	0.001	−0.012	0.017	−0.002	0.148***	−0.034	0.042
	(0.034)	(0.030)	(0.041)	(0.046)	(0.044)	(0.043)	(0.043)
1973–1977	0.056	−0.009	0.113*	0.007	0.281***	0.017	0.047
	(0.037)	(0.033)	(0.045)	(0.050)	(0.048)	(0.047)	(0.046)
1978–82	0.046	−0.016	0.114*	−0.026	0.424***	0.101	−0.003
	(0.044)	(0.039)	(0.052)	(0.059)	(0.056)	(0.054)	(0.054)
1983–1994	0.051	−0.126**	0.101	−0.030	0.475***	0.137*	−0.079
	(0.050)	(0.044)	(0.059)	(0.066)	(0.064)	(0.061)	(0.061)
Time x Age at panel entry	0.004*	0.001	0.003	0.012***	0.003	0.008***	−0.000
	(0.002)	(0.001)	(0.002)	(0.002)	(0.002)	(0.002)	(0.002)
Gender x Time	0.000	−0.000	0.003	−0.002	0.009**	−0.001	−0.007*
	(0.003)	(0.002)	(0.003)	(0.003)	(0.003)	(0.003)	(0.003)
Gender x Age at panel entry	−0.003	0.007	0.051**	0.025	0.038*	0.040*	0.012
	(0.014)	(0.012)	(0.016)	(0.018)	(0.017)	(0.017)	(0.017)
Unemployment rate (%)	−0.030***	−0.019***	−0.018***	−0.021***	−0.025***	−0.019***	−0.021***
	(0.003)	(0.003)	(0.003)	(0.003)	(0.004)	(0.003)	(0.003)
GDP Growth (%)	0.062***	0.069***	0.047***	0.042***	0.047***	0.048***	0.067***
	(0.007)	(0.006)	(0.007)	(0.008)	(0.008)	(0.007)	(0.007)
Constant	6.022***	5.911***	5.429***	4.885***	5.571***	4.943***	5.283***
	(0.030)	(0.027)	(0.034)	(0.038)	(0.038)	(0.035)	(0.035)
S.D.(Slope Time)	0.038***	0.034***	0.039***	0.049***	0.050***	0.050***	0.040***
	(0.003)	(0.002)	(0.003)	(0.003)	(0.003)	(0.002)	(0.002)
S.D.(Intercept)	0.621***	0.537***	0.781***	0.884***	0.815***	0.827***	0.844***
	(0.007)	(0.007)	(0.008)	(0.009)	(0.009)	(0.008)	(0.008)
r(I,S)	−0.260***	−0.207***	−0.168***	−0.145***	−0.291***	−0.126***	−0.289***
	(0.042)	(0.046)	(0.041)	(0.036)	(0.037)	(0.034)	(0.036)
S.D.(Error)	0.644***	0.593***	0.664***	0.724***	0.793***	0.649***	0.626***
	(0.003)	(0.003)	(0.003)	(0.004)	(0.004)	(0.003)	(0.003)
BIC	81,098	74,601	86,413	93,226	96,270	86,798	84,620
AIC	80,903	74,407	86,218	93,032	96,075	86,603	84,425
N	34,129	34,129	34,129	34,129	34,129	34,129	34,129

*Note*. *r*(I,S) = correlation between intercept and slope. *p < 0.05, ** p < 0.01, *** p < 0.001. Standard errors in parentheses. Age, time and value measures are standardized to *Mean* = 0 and *S.D.* = 1.

**Table 11:** Regression table of multilevel models using standardized ratings of value.

	Universalism	Benevolence	Self-direction	Stimulation	Hedonism	Achievement	Conformity
Time	−0.001	−0.021***	−0.018***	−0.024***	−0.013***	−0.025***	−0.012***
	(0.002)	(0.002)	(0.002)	(0.002)	(0.002)	(0.002)	(0.002)
Age at panel entry	0.204***	0.053	0.114**	−0.141***	0.144***	0.008	0.106**
	(0.033)	(0.033)	(0.035)	(0.035)	(0.034)	(0.035)	(0.035)
Gender (ref. Men)	0.307***	0.334***	0.023	−0.132***	0.028	−0.130***	0.183***
	(0.017)	(0.017)	(0.018)	(0.018)	(0.018)	(0.018)	(0.018)
Cohort (Ref. 1964–1968)							
1937–1945	0.106	0.160*	0.007	0.258***	−0.261***	0.193**	0.120
	(0.068)	(0.068)	(0.072)	(0.072)	(0.071)	(0.072)	(0.074)
1946–1949	0.104	0.160**	0.026	0.190**	−0.267***	0.114	0.060
	(0.059)	(0.059)	(0.063)	(0.063)	(0.061)	(0.063)	(0.064)
1950–1952	0.086	0.136*	−0.048	0.088	−0.274***	0.037	−0.008
	(0.054)	(0.054)	(0.057)	(0.058)	(0.056)	(0.058)	(0.059)
1953–1956	0.134**	0.150**	0.017	0.126*	−0.197***	0.090	0.021
	(0.046)	(0.046)	(0.049)	(0.049)	(0.048)	(0.050)	(0.050)
1957–1960	0.105**	0.111**	0.025	0.105*	−0.117**	0.068	−0.016
	(0.040)	(0.040)	(0.044)	(0.044)	(0.042)	(0.044)	(0.045)
1961–1963	0.076*	0.021	−0.014	0.039	−0.064	0.032	−0.023
	(0.038)	(0.038)	(0.041)	(0.042)	(0.040)	(0.042)	(0.042)
1969–1972	0.001	−0.015	0.016	−0.002	0.131***	−0.031	0.040
	(0.037)	(0.037)	(0.040)	(0.040)	(0.039)	(0.040)	(0.041)
1973–1977	0.060	−0.011	0.109*	0.006	0.247***	0.016	0.045
	(0.040)	(0.040)	(0.043)	(0.044)	(0.042)	(0.044)	(0.044)
1978–82	0.050	−0.019	0.110*	−0.023	0.373***	0.094	−0.003
	(0.047)	(0.048)	(0.051)	(0.051)	(0.049)	(0.051)	(0.052)
1983–1994	0.055	−0.153**	0.098	−0.026	0.418***	0.128*	−0.076
	(0.054)	(0.054)	(0.057)	(0.057)	(0.056)	(0.057)	(0.058)
Time x Age at panel entry	0.004*	0.001	0.003	0.010***	0.003	0.008***	−0.000
	(0.002)	(0.002)	(0.002)	(0.002)	(0.002)	(0.002)	(0.001)
Gender x Time	0.000	−0.000	0.003	−0.002	0.008**	−0.000	−0.006*
	(0.003)	(0.003)	(0.003)	(0.003)	(0.003)	(0.003)	(0.003)
Gender x Age at panel entry	−0.003	0.009	0.050**	0.022	0.033*	0.037*	0.011
	(0.015)	(0.015)	(0.016)	(0.016)	(0.015)	(0.016)	(0.016)
Unemployment rate (%)	−0.032***	−0.024***	−0.017***	−0.018***	−0.022***	−0.017***	−0.020***
	(0.003)	(0.003)	(0.003)	(0.003)	(0.003)	(0.003)	(0.003)
GDP Growth (%)	0.067***	0.085***	0.045***	0.036***	0.042***	0.045***	0.064***
	(0.007)	(0.007)	(0.007)	(0.007)	(0.007)	(0.006)	(0.006)
Constant	−0.078*	−0.079*	0.091**	0.179***	0.124***	0.167***	0.003
	(0.032)	(0.033)	(0.033)	(0.033)	(0.033)	(0.033)	(0.033)
S.D.(Slope Time)	0.041***	0.041***	0.038***	0.042***	0.044***	0.047***	0.038***
	(0.003)	(0.003)	(0.002)	(0.002)	(0.003)	(0.002)	(0.002)
S.D.(Intercept)	0.672***	0.656***	0.756***	0.765***	0.717***	0.774***	0.805***
	(0.008)	(0.008)	(0.008)	(0.008)	(0.008)	(0.007)	(0.008)
r(I,S)	−0.260***	−0.207***	−0.168***	−0.145***	−0.291***	−0.126***	−0.289***
	(0.042)	(0.046)	(0.041)	(0.036)	(0.037)	(0.034)	(0.036)
S.D.(Error)	0.696***	0.724***	0.643***	0.626***	0.698***	0.607***	0.597***
	(0.004)	(0.004)	(0.003)	(0.003)	(0.004)	(0.003)	(0.003)
BIC	86,433	88,221	84,217	83,310	87,521	82,233	81,402
AIC	86,239	88,027	84,022	83,116	87,326	82,038	81,208
N	34,129	34,129	34,129	34,129	34,129	34,129	34,129

*Note*. *r*(I,S) = correlation between intercept and slope. *p < 0.05, ** p < 0.01, *** p < 0.001. Standard errors in parentheses. Age, time and value measures are standardized to *Mean* = 0 and *S.D.*

**Table 12:** Value definitions from the theory of basic human values.

Value	Definition
Self-direction	Independent thought and action—choosing, creating, exploring
Stimulation	Excitement, novelty, and challenge in life
Hedonism	Pleasure and sensuous gratification for oneself
Achievement	Personal success through demonstrating competence according to social standards
Power	Social status and prestige, control or dominance over people and resources
Security	Safety, harmony, and stability of society, of relationships and of self
Conformity	Restraint of actions, inclinations and impulses likely to upset or harm others and violate social expectations or norms
Tradition	Respect, commitment, and acceptance of the customs and ideas that traditional culture or religion provides
Benevolence	Preservation and enhancement of the welfare of people with whom one is in frequent social contact
Universalism	Understanding, appreciation, tolerance and protection for the welfare of all people and nature.

*Note.* Adapted from Schwartz, S. H., Cieciuch, J., Vecchione, M., Davidov, E., Fischer, R., Beierlein, C., Ramos, A., Verkasalo, M., Lönnqvist, J.-E., Demirutku, K., Dirilen-Gumus, O., & Konty, M. (2012). Refining the theory of basic individual values. Journal of Personality and Social Psychology, 103(4), 663–688. https://doi.org/10.1037/a0029393

## Footnotes

1 All respondents answered the RVS in 2008 and 2009 after which it was fielded every two years. However, several refreshment samples also answered the RVS when entering the panel. Additionally, the timing of the personality module changed from June to November in 2014 and 2015.

2 That is, the first time that an individual was observed and answered the RVS was T_0_ and coded as time = 0. The calendar year could be 2008 for those initially recruited or 2010, 2012 or 2014 for those participants from the respective refreshment samples. For each person-year we then calculated the number of days that elapsed since the T_0_ and transformed the variable into a year measure.
